# Miniscrew-assisted rapid palatal expander (MARPE): the quest for pure orthopedic movement

**DOI:** 10.1590/2177-6709.21.4.017-023.oin

**Published:** 2016

**Authors:** Hideo Suzuki, Won Moon, Luiz Henrique Previdente, Selly Sayuri Suzuki, Aguinaldo Silva Garcez, Alberto Consolaro

**Affiliations:** 1 Professor, responsible for the Masters Program in Orthodontics, São Leopoldo Mandic, School of Dentistry, Campinas, São Paulo, Brazil.; 2 Associate clinical professor and director of the Residency Program of Orthodontics, University of California, Los Angeles, USA.; 3 Professor, São Leopoldo Mandic, School of Dentistry, Graduate Program, Campinas, São Paulo, Brazil.; 4 Professor, São Leopoldo Mandic, School of Dentistry, Masters Program in Orthodontics, Campinas, São Paulo, Brazil.; 5 Professor of Microbiology, São Leopoldo Mandic, School of Dentistry, Campinas, São Paulo, Brazil.; 6 Full professor, Universidade de São Paulo (USP), School of Dentistry, Department of Orthodontics, Bauru, São Paulo, Brazil. Professor, Universidade de São Paulo (USP), School of Dentistry, Graduate Program, Ribeirão Preto, São Paulo, Brazil.

**Keywords:** Midpalatal suture, Rapid palatal expansion, Palatal expansion, MARPE.

## Abstract

The midpalatal suture has bone margins with thick connective tissue interposed between them, and it does not represent the fusion of maxillary palatal processes only, but also the fusion of palatal processes of the jaws and horizontal osseous laminae of palatal bones. Changing it implies affecting neighboring areas. It has got three segments that should be considered by all clinical analyses, whether therapeutic or experimental: *the anterior segment* (before the incisive foramen, or intermaxillary segment), *the middle segment* (from the incisive foramen to the suture transversal to the palatal bone ) and *the posterior segment* (after the suture transversal to the palatal bone ). Rapid palatal expansion might be recommended for patients at the final pubertal growth stage, in addition to adult patients with maxillary constriction. It represents a treatment solution that can potentially avoid surgical intervention. When performed in association with rapid palatal expanders, it might enhance the skeletal effects of the latter. Of the various designs of expansion appliances, MARPE (miniscrew-assisted rapid palatal expander) has been modified in order to allow its operational advantages and outcomes to become familiar in the clinical practice.

## RESUMO

A sutura palatina mediana tem margens ósseas interpostas por tecido conjuntivo denso e não representa apenas a união dos processos palatinos da maxila, mas também a dos processos alveolares das maxilas e das lâminas horizontais dos ossos palatinos. Modificá-la implica em influenciar outras áreas vizinhas. Ela tem três segmentos a serem considerados em todas as análises clínicas, terapêuticas e experimentais: *anterior* (antes do forame incisivo, ou intermaxilar), *médio* (do forame incisivo até a sutura transversa com o osso palatino) e *posterior* (após a sutura transversa com o osso palatino). A expansão rápida da maxila pode ser indicada para pacientes que se encontram no final da fase de crescimento da puberdade, além de pacientes adultos com atresia maxilar, representando uma solução de tratamento, possivelmente evitando uma intervenção cirúrgica. Quando associada a aparelhos de protração maxilar, pode maximizar os efeitos esqueléticos desses. Entre os vários *designs* de aparelhos disjuntores, ultimamente encontra-se o MARPE (*Miniscrew-Assisted Rapid Palatal Expander*), que tem sido modificado para que ocorra, na rotina clínica, uma familiarização com suas vantagens operatórias e resultados obtidos.

Both the macroscopic and microscopic morphologies of the midpalatal suture were described by Ennes,^13^ Ennes et al^12^ and Ennes and Consolaro^14^ in 2004 and 2004, including its morphological changes in terms of the chronological evolution of humans, primates, rabbits and rats.

Understanding the biological events implicated in orthodontic, orthopedic and surgical procedures carried out in the midface requires knowledge about the structures of the midpalatal suture vertically and horizontally at different age groups. 

**Figure 1:**
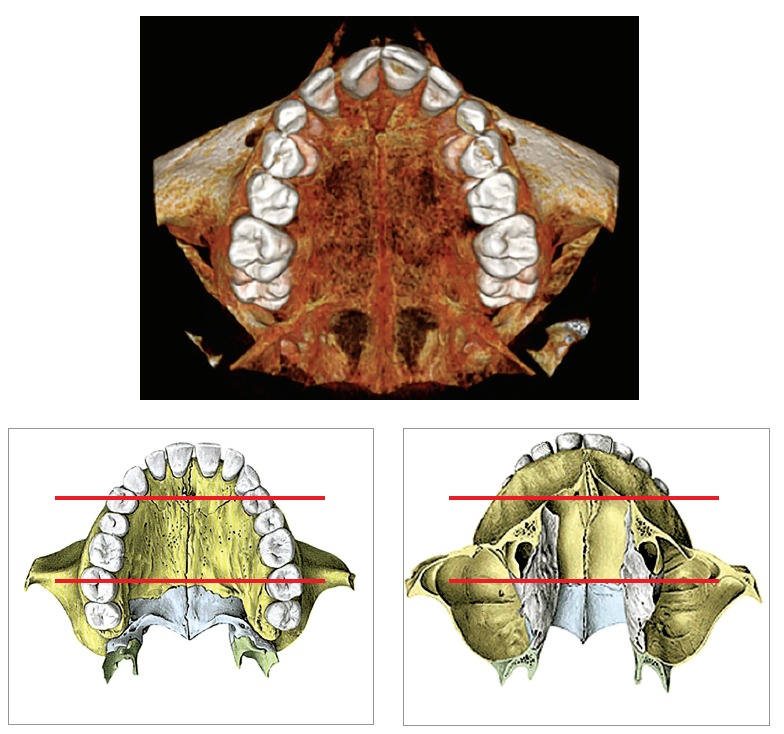
Midpalatal suture and its three segments (anterior, middle and posterior) which are relative to other structures, for instance, the transverse palatine suture, and nasal bones.

The midpalatal suture is wrinkled and arranged in an overlapping as well as sinuous pattern, with bone margins with thick connective tissue interposed between them in three to five layers. It should be highlighted that the midpalatal suture:1) Does not represent the fusion of maxillary palatal processes only, but also the fusion of alveolar palatal processes of the jaws and horizontal osseous laminae of palatal bones. Changing it implies affecting neighboring areas (Fig 1).

2) It has got three segments (Fig 1) that should be considered by all clinical analyses, whether therapeutic or experimental:

» *the anterior segment:* before the incisive foramen, or intermaxillary segment;
» *the middle segment:* from the incisive foramen to the suture transversal to the palatal bone;
» *the posterior segment:* after the suture transversal to the palatal bone.

Morphological and clinical-therapeutic approaches often aim at the midpalatal suture, but do not include its anterior segment. Likewise, they occasionally aim at its posterior segment.

**Figure 2:**
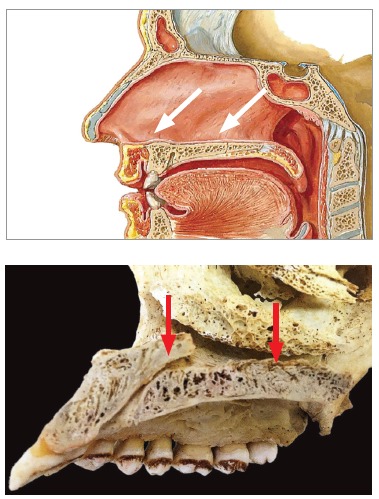
Midpalatal suture: note that the incisive canal distinguishes the anterior and middle segments. It goes in posterior and upward direction. The incisive canal has got vessels, nerves, salivary glands and nasopalatine canal remnants. The posterior segment is relative to the suture transversal to the palatal bone.

The influence exerted by rapid maxillary expansion (RME) over other structures relative to the midpalatal suture is rarely carefully studied.^31^ To what extent does the suture (Figs 1 and 2) transversal to the palatal bone lessen forces applied by maxillary expansion appliances, given that it represents an interruption of a continuous solid structure?

The palate develops from the sixth to the 12^th^ week of embryonic life. The primary palate is the secondary ossification center of the maxilla, arising from the medial nasal processes in the intermaxillary segment. During the eighth week of intrauterine development, ossification centers merge to form the maxilla.^19^ The primary palate establishes a palatine view of the suture between the premaxilla and the maxilla with the secondary palate,^27^ without leaving any signs or marks in the adult individual. The same process does not apply to the palatal bone with which the suture is well established.

The osteogenic process in the midpalatal suture is similar to the growth and bone remodeling provided by the periosteum in other bone surfaces, and fulfills the requirements of adjacent tissues by means of external stimuli also known as functional demands.^9^ The midpalatal suture is not a center of maxillary bone growth,^11^ ^,^ ^20^ ^,^ ^25^ but it responds to osteogenic stimuli.

## MIDPALATAL SUTURE CHRONOLOGY AS PER AGE GROUP

The chronological developmental phases of ossification of the human midpalatal suture were microscopically studied in 24 individuals, aged between 15 and 35 years old, by Persson and Thilander^29^ in 1977. Ossification of the suture starts at the posterior region by means of mineralized bridges formed from posteriorly to anteriorly, varying according to the chronological age at which they form - in consonance with the end of the growth stage and facial development, under influence exerted by bone maturation.

After analyzing the skeletal age of the palatal sutures of 186 human skulls, Mann et al^2^ identified, in 1991, the following sequence of ossification:

1. It starts with the incisive suture.
2. Followed by the posterior segment of the midpalatal suture.
3. Followed by the transverse palatine suture.
4. Finally, it is followed by the middle segment of the midpalatal suture.

Ossification of the suture has been considered as a limiting factor for rapid palatal expansion, and it seems to be a consensus that it starts at the posterior segment.^32^

Imaging diagnosis of closure or ossification of the midpalatal suture remains limited^2^ ^,^ ^12^ ^,^ ^13^ ^,^ ^14^ in occlusal radiographs or tomographic slices, especially if we consider that the small bridges formed are microscopically identified, but do not necessarily result in visible images. Small bone length and/or thickness cannot be detected by imaging examination, especially if calibration and resolution of modern appliances are considered.

Despite the advances made in the last ten years, the conclusions reached by Ennes,^13^ Ennes et al^12^ and Ennes and Consolaro,^14^ in 2002 and 2004, with studies conducted on the morphology of the midpalatal suture at different stages of development, remain appropriate, especially regarding the following:

1) By using modern diagnostic tools, it is impossible to precisely and safely perform clinical and radiographic analyses of the onset of ossification of the midpalatal suture and its degree of structural implication. It is possible to claim that the older the patient, the higher the likelihood that the midpalatal suture and maxillary inter-relations become ossified.

2) In human beings, ossification of the midpalatal suture occurs within the period from adulthood to the elderly stages in life; however, the primary fragility of ossification bridges is likely not to allow us to render them responsible for the unsuccessful outcomes of some intermaxillary expansion procedures. Should that be the case, it is recommended that factors inherent to technique and ossification of other facial sutures be reassessed.

In other words, the quest for new means and appliances aimed at performing palatal expansion must be encouraged, so that we can increasingly make advances in this technique in favor of the best results for patients.

## RAPID PALATAL EXPANSION AND MARPE

The first palatal expansion appliance was developed by Angell^3^ in 1860. At that time, the procedure was not incorporated to the orthodontic practice, as it was considered inappropriate by Angell's colleagues who were influenced by rhinologists' fears. 

European orthodontists, Maxillary Orthopedics enthusiasts, brought the technique back based on the works by Derischsweiler (1953) and Korkhaus^18^ (1960). American Orthodontics became interested in it when Haas,^15^ in 1961, carried out the procedure in pigs and proved the existence of the microscopic events implicated. The technique employed in patients with atrophic maxilla achieve positive outcomes and the procedure was considered safe and as an alternative for more complicated cases, such as Class II malocclusion associated with posterior crossbite. 

From that time onwards, other appliance designs were proposed with the same purpose: as an alternative to correct malocclusions associated with atrophic maxilla. If the patient performs 2/4 of a turn every day, expansion occurs within one to two weeks. The desired extension is achieved, including overcorrection and relapse. The latter often occurs as a result of lack of balance or tensegrity among the pieces that form the midface.^7^

The orthopedic expansion appliance - with or without a screw secured to a resin support adapted to the palatal mucosa - exerts force onto supporting teeth, thereby leading to a decrease of blood flow in the buccal periodontium while also forming extensive hyaline areas^8^ in which the conditions for the recovery of bone modeling units, essential for tooth movement to occur within the bone, are nonexistent. Bone resorption occurs at a distance and at a slower pace, keeping the supporting teeth in position until the suture yields to the resultant force. 

Once expansion has been evinced, by means of diastema opening between maxillary central incisors, the next step is to achieve crossbite overcorrection, so as to prevent relapse. At this stage, the expansion appliance is inactivated for three months. After this period, a retention is installed and kept for six months. In cases in which maxillary expansion is not successfully achieved by means of orthodontic procedures, surgery might be recommended for rapid palatal expansion. 

Undesirable effects include discomfort at the regions of incisors or nasal suture, and ulceration or necrosis of the palatal mucosa. There might be some swelling at the midpalatal suture, particularly soon after expansion. Ischemia and necrosis of the palatal mucosa might occur when the suture does not yield to forces applied by the tooth-mucosa-supported appliance.^30^ ^,^ ^34^ The mucosal lesion resulting from decreased blood flow - caused by the resultant force - make the procedure unfeasible in the event of occurring before expansion. Supporting teeth might undergo tooth resorption on the buccal surface of roots.^8^ ^,^ ^28^

The outcomes of palatal expansion vary from failure to a horizontal gain of 4 mm. Failure has been attributed to: patient's skeletal maturity;^36^ ^,^ ^37^ variation in the transverse measure, depending on the post-treatment moment when data were collected; and retention,^33^ with relapse of horizontal measures and maxillary movement downward being identified - with the maxilla, later on, remaining unchanged or with its primary position restored.^4^ ^,^ ^5^ ^,^ ^6^ ^,^ ^17^ ^,^ ^37^

In order to increasingly enhance the procedure of palatal expansion, one seeks to improve or innovate the appliances used. In 2010, Lee et al^21^ treated a 20-year-old patient with severe transverse discrepancy and mandibular prognathism. Before orthognathic surgery, the patient used an expansion appliance secured to the palate by means of miniscrews (miniscrew-assisted rapid palatal expander, or MARPE). Expansion was achieved with minimal damage to teeth and periodontium, with stable outcomes confirmed by clinical and radiographic examination. The authors concluded that it is an effective treatment modality used for transverse correction and which might eliminate the need for a few surgical procedures in patients with craniofacial discrepancies, thus taking advantage of the possibilities offered by the sutures.

**Figure 3:**
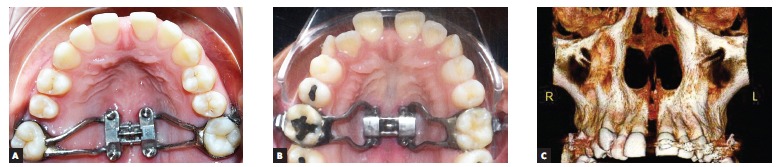
MARPE appliance in which miniscrews are incorporated to the screw support design, with measures determined on the basis of morphology of the palatal region parallel to the midpalatal suture: A) MSE expansion appliance (maxillary skeletal expander, Biomaterial Korea, Seul, South Korea, developed by Moon et al^26^ ^,^ ^35^); B) MARPE appliance modified by Suzuki et al^10^ ^,^ ^35^ (Peclab, Belo Horizonte/MG, Brazil). C) computed tomography after expansion (in B).

Recently, based on Lee's studies, Park and Hwang,^10^ ^,^ ^21^ Moon^26^ and MacGinnis et al^23^ developed the maxillary skeletal expander (MSE, Biomaterial Korea, Seoul, South Korea) with four miniscrews installed into the expansion screw body, parallel to the midpalatal suture and to itself. Even more recently, Suzuki et al^35^ changed the rapid maxillary expansion appliance, securing it by means of miniscrews (MARPE); however, with a different design (Peclab, Belo Horizonte, Brazil) (Fig 3). MARPE's new design has been used in a number of patients with atrophic maxilla, both young, growing patients and adult ones. 

In the appliance developed by Lee et al,^21^ the miniscrews are secured to the turn-key by means of extensions welded to the expansion screw, and joined with light-curing resin. With miniscrews kept away from the midpalatal suture, there is an increase in the risk of perforating underlying structures (such as canals and nerves in both anterior and posterior regions), as well as on the sides, which is even more serious, as there would be four sites to be chosen individually. Alves et al^1^ mapped the areas of risk implied in securing miniscrews onto the human palate. In MSE^26^ ^,^ ^35^ and MARPE^10^ ^,^ ^35^ appliances, miniscrews are used as a support for the expansion screw (Fig 3) and would be secured in a more even manner parallel to the suture, with a view to aiming at a thicker bone area, so as to increase primary stability and provide a more efficient propagation of forces to the nasomaxillary complex.

The midpalatal suture is located right behind the incisive foramen, which represents the mouth of a canal that goes up in posterior direction. It might have an opening at the nasal cavity, as high as the line tangent to the distal surfaces of both maxillary canines (Fig 2). The risk of screws affecting this structure is little, although this might occasionally happen. 

**Figure 4:**
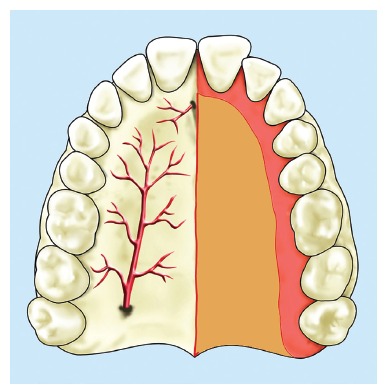
Vessels and nerves in the palatal region, relative, directly or indirectly, to the midpalatal suture. In the region of canines, a plexus comprising those structures are formed on the submucosa.

Likewise, at canines height, in the lateral palatal region, there is the nerve and blood vessel plexus, with anastomosis between vessels coming out from the incisive foramen and, in forward direction, from the palatine foramina (Fig 4). 

Meanwhile, in the posterior hard palate, there is a transverse suture between maxillary palatine processes and the horizontal osseous laminae of the palatal bone. Miniscrews secured too posteriorly might be located at that structure. Although the latter does not undergo natural movement, it is a fibrous connective tissue under remodeling influence due to the expanding procedure. In this context, adding the fixing presence of a miniscrew might not be convenient, although there have been no studies assessing potential biological inconvenience.

In continuity with the soft palate, the posterior hard palate has a number of small salivary glands. Miniscrews placed too posteriorly might affect such glands, thus provoking mucus-retention phenomena - similarly to what occurs with oral mucocele and/or necrotizing sialometaplasia.^30^ At the posterior lateral portion of the hard palate, near the posterior alveolar process, there are nerves and vessels which emerge from the palatine foramina (Fig 4).

Placing miniscrews away from the body of the expansion screw allows a more effective use; however, with greater risks. Miniscrews might be contextualized and become part of orthodontic and orthopedic treatment carried out with elastics and wires, in addition to being useful as anchorage units. Whenever they are internalized, as in the design by Suzuki et al^10^ ^,^ ^35^ (Fig 3B), those possibilities are not applicable.

The miniscrew-assisted rapid palatal expander (MARPE) is characterized by a decrease in the excessive load performed by conventional appliances onto the buccal periodontal ligament of teeth to which they are anchored, thus resulting in flat, multiple resorption on their roots. However, clinically speaking, it does not involve any risks to patients. There is also a considerable decrease in the accidental movement of anchoring teeth, given that, with the use of MARPE, the support for the palatal expansion is no longer dental, but osseous.

More recent studies have recommended MARPE to treat growing patients with transverse and anteroposterior maxillary deficiency and recommendation of maxillary protraction. MARPE miniscrews would enhance the skeletal effects produced by maxillary advancement, as they are anchored at the basal bone of the maxilla, thus resulting in pure orthopedic movement while minimizing the effects produced on teeth.^22^

## FINAL CONSIDERATIONS

Rapid palatal expansion might be recommended for patients at the final pubertal growth stage, in addition to adult patients with maxillary constriction. It represents a treatment solution that can potentially avoid surgical intervention. When performed in association with rapid palatal expanders, it might enhance the skeletal effects of the latter. 

The MARPE appliance modified by Suzuki et al^10^ ^,^ ^35^ aims at making its use appropriate to the clinical practice, so that operational advantages and outcomes are rendered familiar to all.
